# Biomechanical investigation of a minimally invasive posterior spine stabilization system in comparison to the Universal Spinal System (USS)

**DOI:** 10.1186/s12891-016-0983-1

**Published:** 2016-03-22

**Authors:** D. Kubosch, E. J. Kubosch, B. Gueorguiev, I. Zderic, M. Windolf, K. Izadpanah, N. P. Südkamp, P. C. Strohm

**Affiliations:** Department of Orthopaedic and Trauma Surgery, University of Freiburg Medical Center, Hugstetterstr. 55, D-79106 Freiburg im Breisgau, Germany; AO Research Institute Davos, Clavadelerstrasse 8, CH-7270 Davos, Switzerland

**Keywords:** Fracture, Biomechanic, Minimally invasive surgery, Percutaneous fixation, Pedicle screw, Polyaxial

## Abstract

**Background:**

Although minimally invasive posterior spine implant systems have been introduced, clinical studies reported on reduced quality of spinal column realignment due to correction loss. The aim of this study was to compare biomechanically two minimally invasive spine stabilization systems versus the Universal Spine Stabilization system (USS).

**Methods:**

Three groups with 5 specimens each and 2 foam bars per specimen were instrumented with USS (Group 1) or a minimally invasive posterior spine stabilization system with either polyaxial (Group 2) or monoaxial (Group 3) screws.

Mechanical testing was performed under quasi-static ramp loading in axial compression and torsion, followed by destructive cyclic loading run under axial compression at constant amplitude and then with progressively increasing amplitude until construct failure.

Bending construct stiffness, torsional stiffness and cycles to failure were investigated.

**Results:**

Initial bending stiffness was highest in Group 3, followed by Group 2 and Group 1, without any significant differences between the groups.

A significant increase in bending stiffness after 20’000 cycles was observed in Group 1 (*p* = 0.002) and Group 2 (*p* = 0.001), but not in Group 3, though the secondary bending stiffness showed no significant differences between the groups.

Initial and secondary torsional stiffness was highest in Group 1, followed by Group 3 and Group 2, with significant differences between all groups (*p* ≤ 0.047). A significant increase in initial torsional stiffness after 20’000 cycles was observed in Group 2 (*p* = 0.017) and 3 (*p* = 0.013), but not in Group 1.

The highest number of cycles to failure was detected in Group 1, followed by Group 3 and Group 2. This parameter was significantly different between Group 1 and Group 2 (*p* = 0.001), between Group 2 and Group 3 (*p* = 0.002), but not between Group 1 and Group 3.

**Conclusions:**

These findings quantify the correction loss for minimally invasive spine implant systems and imply that unstable spine fractures might benefit from stabilization with conventional implants like the USS.

## Background

Spinal fractures account for approximately 6 % of all skeletal injuries [1]. With 35 % of all cases, the most frequent cause of such fractures is a fall from a height, leading to compression of the vertebral bodies as a result of acting excessive axial forces [2–4]. Thus, the majority of traumatic injuries of the spinal column are related to compression fractures, classified as Type A by Magerl [4], in contrast to distraction (Type B) and rotation (Type C) injuries. However, it is not only the direction of the applied force, but also the energy absorbed by the vertebral body, that determines the type and severity of the injury [5]. The thoracolumbar junction is most frequently affected in 67 % of the spine fractures, followed by injuries of the thoracic and the lumbar spine in 18 and 13 % of the cases, respectively. Most of the affected patients are between 20 and 40 years old, and predominantly male (66.4 %) [3, 4].

The most appropriate way to treat spine compression fractures still remains subject of controversial discussion. Surgical intervention offers an alternative to conservative corset treatment with options including purely posterior and purely anterior surgical approaches, as well as a combined antero-posterior procedure [6].

The management of a spinal fracture should aim at restoration of the correct/native sagittal and frontal profiles [7]. Particularly in cases of compression fractures in the region of the thoracic and lumbar spine, a common approach to achieve this objective is posterior instrumentation with fixed-angle implant systems such as the Universal Spine System (USS, DePuy Synthes, Zuchwil, Switzerland). It has therefore become the wide spread standard for management of vertebral body fractures. If necessary, the technique can be combined with anterior fusion involving implantation of different types of cages and/or tricortical bone transplants [8].

The main advantage of a conventional open approach is that decompression of the spinal canal and sufficient distraction of the spinal column can be achieved simultaneously. Decompression and realignment of the vertebral bodies can be performed by means of ligamentotaxis. The main disadvantage of the open surgery is the relatively high approach-related morbidity and associated traumatization of the autochthonous muscles of the back.

Minimally invasive techniques and implant systems to perform spondylodesis of a motion segment of the spine appear to be gaining more widespread acceptance in clinical routine due to their obvious advantages over conventional open procedures [9, 10]. Having been utilized in orthopaedic surgery for several years, these implants are nowadays predominantly indicated for treatment of degenerative disease related to segmental instabilities of the spine. In addition, percutaneous minimally invasive stabilization systems have also been increasingly used for management of thoracolumbar fractures and other traumatic injuries of the vertebral bodies for a few years [11]. Such systems may require insertion of mono-, polyaxial, and unilateral screw combinations.

The outcomes reported in the literature for minimally invasive spondylodesis are promising, but the follow-ups are so far very short [12]. However, despite the short follow-up intervals, loss of the intraoperatively achieved vertebral body realignment and restored sagittal profile is often described [13]. Therefore, a biomechanical comparison of monoaxial and polyaxial minimally invasive systems versus conventional open stabilization with fixed-angle implants is necessary [11].

Regarding the existing minimally invasive spine implant systems, it is frequently hypothesized in the literature that the polyaxial or monoaxial screws offer insufficient biomechanical stability to maintain long-term intraoperative reduction [13–15]. Therefore, the aim of this study was to investigate biomechanically a minimally invasive spine posterior stabilization system with monoaxial or polyaxial screws in comparison to USS in terms of axial and torsional stiffness, and cycles to failure.

## Methods

The current study was approved by the AOTRAUMA Research Commission.

### Specimen preparation

Thirty identical solid rigid polyurethane foam bars (40 pcf) (Sawbones Europe AB, Malmö, Sweden) in the shape of a rectangular parallelepiped with dimensions 40 mm × 40 mm × 30 mm, density 0.64 g/cm^3^, elastic modulus 1.19GPa, shear modulus 0.187GPa and ultimate tensile strength 16 MPa, representing artificial vertebra, have been used in this study. They were divided into three study groups with 5 specimens each and 2 bars per specimen, and instrumented with either a conventional USS implant (Group 1) for dorsal spondylodesis (DePuy Synthes, Zuchwil, Switzerland), or a Globus Revolve implant (Globus Medical, Audubon, USA) with either polyaxial (Group 2) or monoaxial (Group 3) screws according to the manufacturers’ guidelines as described below. A minimum of 6 specimens was required as a sample size per group to achieve statistical power of 0.8 at a level of significance 0.05.

**Fig. 1:**
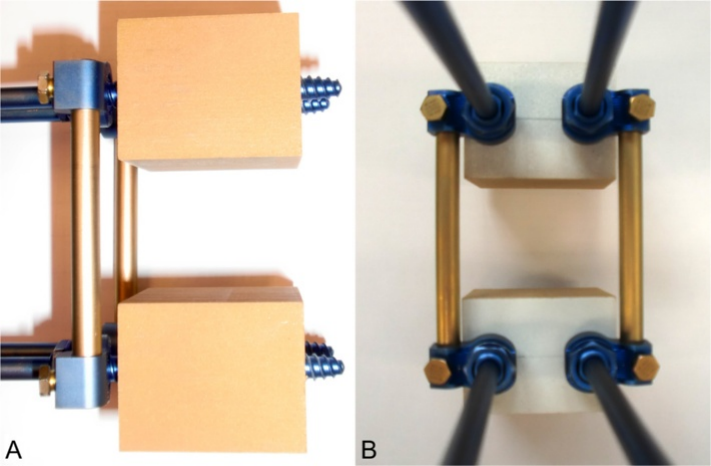
Solid rigid polyurethane foam bars instrumented with USS (screw: diameter 6.2 mm, length 50 mm; longitudinal rod: diameter 6 mm, length 70 mm). Left (**a**) lateral view; right (**b**) posterior view

**Fig. 2:**
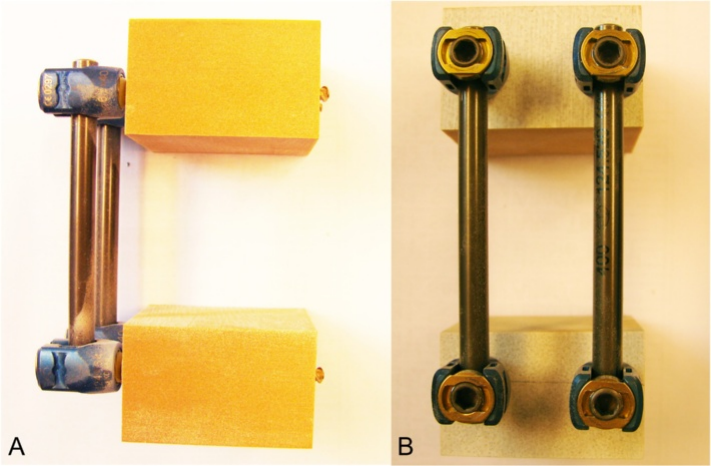
Solid rigid polyurethane foam bars instrumented with Globus revolve polyaxial (screw: diameter 6.5 mm, length 40 mm; longitudinal rod: diameter 5 mm, length 70 mm). Left (**a**) lateral view; right (**b**) posterior view

**Fig. 3:**
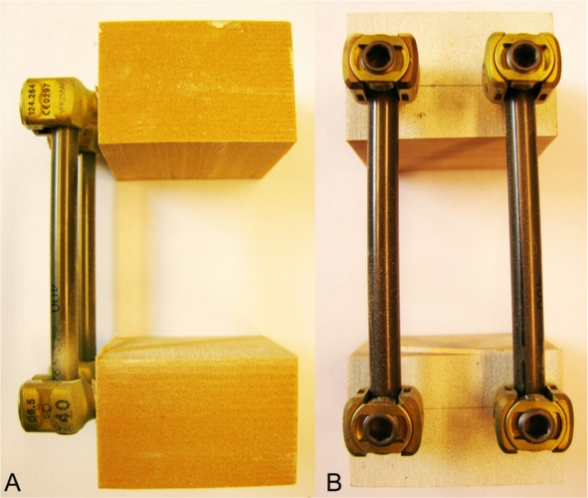
Solid rigid polyurethane foam bars instrumented with Globus revolve monoaxial (screw: diameter 6.5 mm, length 40 mm; longitudinal rod: diameter 5 mm, length 70 mm). Left (**a**) lateral view; right (**b**) posterior view

A mono-segmental dorsal spondylodesis over two levels was performed in all study groups similarly as in the clinical routine, representing a motion segment. Firstly, two screws were placed in each of the two foam bars dorsally, converging along the length of 40 mm at an angle of 10 to 15°. Then the respective screw heads were connected via two rods for each specimen so that the two bars were fixed at a distance of 40 mm from each other as shown in Figs. 1, 2 and 3 for Group 1, 2 and 3, respectively.

### Mechanical testing

**Fig. 4:**
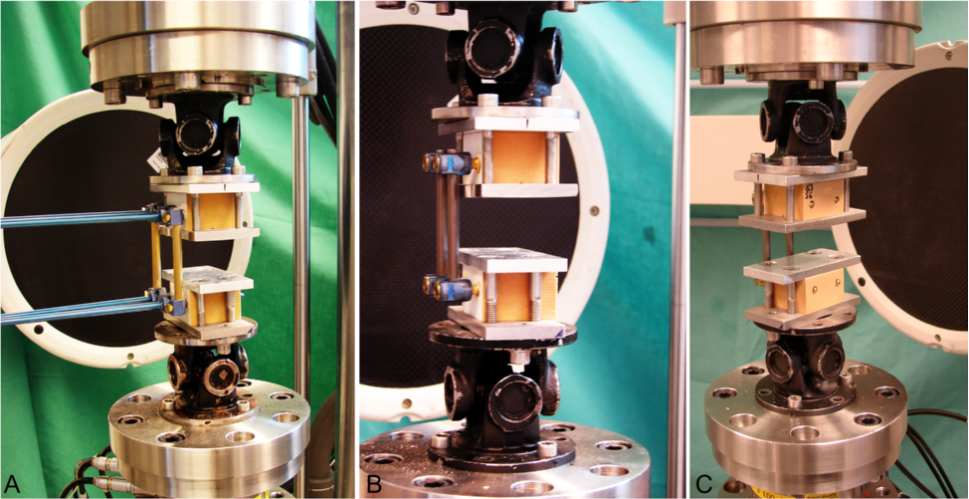
Setup with specimens mounted for biomechanical testing after instrumentation with USS (**a**), Globus revolve polyaxial (**b**) and Globus revolve monoaxial (**c**) [29]. Each specimen is positioned in the machine axis and attached to the machine actuator (top) and the load cell (bottom) with two cardan joints after clamping of the foam bars between two metal plates

Mechanical testing was performed on a biaxial servo-hydraulic machine MTS Mini Bionix II 858 (MTS Systems Corp., Eden Prairie, MN, USA) with a 4kN/20 Nm load cell. The specimens were attached to the machine actuator and the load cell, the latter restrained to the machine frame, by means of two cardan joints (Fig. 4a-c). For this purpose, each of the specimen foam bars was clamped between two metal plates and fixed to the respective cardan joint with four bolts so that its center was positioned in the machine axis. The use of two cardan joints allowed free anteroposterior and sagittal bending of the two foam bars with respect to their connections to the machine components during the load transfer.

The loading protocol comprised a quasi-static and a cyclic loading part. The quasi-static loading was performed at the beginning and repeated after 20’000 cycles (of the cyclic loading part) to investigate the elastic behaviour of each specimen at these two time points. It consisted of a non-destructive axial compression ramp between 50 and 350 N at a rate of 30 N/s, repeated three times to consider settling effects, followed by a non-destructive torsional ramp ±4 Nm with a compressional preload of 50 N repeated also three times at a rate of 0.4 Nm/s, starting from 0 Nm.

The cyclic mechanical test was performed at a rate of 2 Hz with sinusoidal axial loading at a constant amplitude of 300 N during the first 20’000 cycles, keeping the axial cyclic compression forces within a range of 50 N (valley) to 350 N (peak). After 20’000 cycles, the peak level was progressively increased, starting from 350 N, at a rate of 80 mN/cycle until construct failure. The loading protocol for cyclic testing simulated patient activities as a combination of a limited range of movements under invariable loading for spine protection during the initial postoperative phase, and a later steady increase of loading during rehabilitation and healing. It was defined in a good agreement with some biomechanical data from the literature [16–18]. The principle of cyclic testing with progressively increasing load levels has proven to be useful in previous studies [19].

### Data acquisition and analysis

Axial displacement, axial load, angle and torque were recorded during the mechanical tests from the machine’s transducers at a sampling rate of 128 Hz.

Based on the machine data from the quasi-static tests at the beginning and after 20’000 cycles, axial bending and torsional construct stiffness were calculated from the load–displacement and torque-angle curves using Matlab software package (The MathWorks, Natick, Massachusetts, USA).

At the beginning of the cyclic test and then every 1’000 cycles until failure, the machine actuator stopped for 2 s in unloading condition at 50 N valley load in order to perform a lateral fluoroscopic assessment with a C-arm to associate the loading history with the specimen’s plastic deformation in flexion. A relative 5° increase of specimen flexion at the valley load in comparison to the initial specimen condition was defined as the arbitrary failure criterion and the number of cycles to reach this angulation, defined as cycles to failure, were derived from the radiographs.

Statistical analysis was performed using SPSS software package (IBM SPSS, Chicago, IL, USA). Normal distribution and homogeneity of variances were tested with the Shapiro-Wilk test and Levene test, respectively. For detection of significant differences between the study groups regarding the axial bending stiffness, torsional stiffness and cycles to failure, One-Way Analysis of Variance (ANOVA) with Bonferroni PostHoc multiple comparisons was applied. The evolution of the initial axial bending stiffness and torsional stiffness in each study group after 20’000 cycles was analysed with paired T-Test. The significance level was set to 0.05 for all statistical tests.

## Results

All parameters of interest taken for statistical evaluation were normally distributed in each of the three study groups and with homogeneity of variance between the groups.

**Fig. 5:**
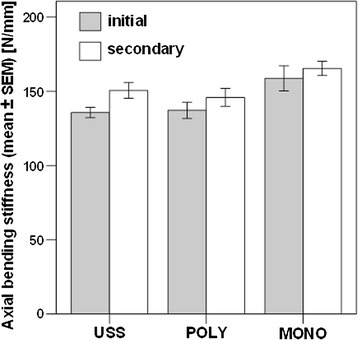
Bar plots representing initial and secondary axial bending stiffness in the three study groups instrumented with USS, polyaxial (POLY) and the monoaxial (MONO) screws

The initial axial bending stiffness was highest in Group 3 (Revolve monoaxial, mean ± SEM: 158.72 ± 8.47 N/mm), followed by Group 2 (Revolve polyaxial, 137.19 ± 5.49 N/mm) and Group 1 (USS, 136.68 ± 3.47 N/mm), with no significant differences between the groups. Significant increase of initial axial bending stiffness after 20’000 cycles was observed in Group 1 (*p* = 0.002) and Group 2 (*p* = 0.001), but not in Group 3, whereas the corresponding values for secondary axial bending stiffness after 20’000 cycles (Group 1: 150.57 ± 5.31 N/mm, Group 2: 145.90 ± 6.08 N/mm, Group 3: 165.41 ± 4.78 N/mm) showed no significant differences between the groups (Fig. 5).

**Fig. 6:**
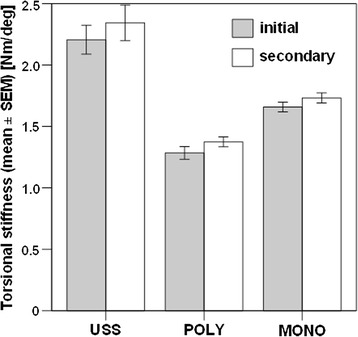
Bar plots representing initial and secondary torsional stiffness in the three study groups instrumented with USS, polyaxial (POLY) and the monoaxial (MONO) screws

Initial and secondary torsional stiffness (the latter after 20’000 cycles) was highest in Group 1 (2.21 ± 0.12 Nm/deg and 2.34 ± 0.14 Nm/deg, respectively), followed by Group 3 (1.65 ± 0.04 Nm/deg and 1.73 ± 0.04 Nm/deg) and Group 2 (1.28 ± 0.05 Nm/deg and 1.37 ± 0.04 Nm/deg) with significant differences between all groups (Table 1). Significant increase of the initial torsional stiffness after 20’000 cycles was observed in Group 2 (*p* = 0.017) and Group 3 (*p* = 0.013), whereas only a trend to significance for this increase was detected in Group 1 (*p* = 0.052) (Fig. 6).

**Table 1:** *P*-values, showing significant differences within all pairs of study groups, formed between Group 1 (USS), Group 2 (POLY) and Group 3 (MONO), with regard to the initial and secondary torsional stiffness (the latter after 20,000 cycles)

Group pairs	Torsional stiffness, Nm/deg	
	Initial	Secondary
USS-MONO	0.002	0.002
USS-POLY	0.001	0.001
MONO-POLY	0.015	0.047

**Fig. 7:**
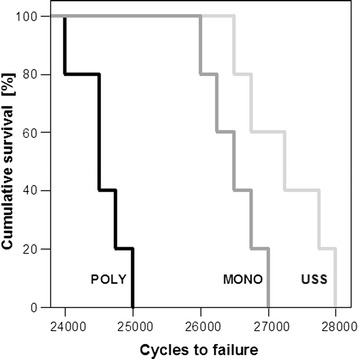
Cumulative survival plots representing cycles to failure in the three study groups instrumented with USS, polyaxial (POLY) and the monoaxial (MONO) screws

The highest number of cycles to failure was observed in Group 1 (27’250 ± 285), followed by Group 3 (26’550 ± 184) and Group 2 (24’550 ± 166). This parameter was significantly different between Group 1 and Group 2 (*p* = 0.001), Group 2 and Group 3 (*p* = 0.002), but not between Group 1 and Group 3 (Fig. 7).

## Discussion

Posterior stabilization is currently the standard procedure for surgical treatment of fractures of the thoracic and lumbar spine in most hospitals and clinics. It is also frequently performed to manage other disorders of the spine, eg. oncotic disorders or inflammatory changes, such as spondylitis [20].

The more frequent application of minimally invasive techniques and implant systems for posterior stabilization of the spine reflects several advantages of this method, such as less intraoperative blood loss, reduced postoperative pain, and a shorter hospital stay [10, 20, 21]. This is particularly important in the context of demographic change in industrial countries since the comorbidities of an aging patient collective must be integrated into the overall treatment plan [22].

Despite these advantages, it is important to carefully evaluate the use of minimally invasive spine systems, due to their still existing disadvantages to conventional open procedures, such as limited scope for distraction, which have to be considered critically during treatment of compression fractures [23].

Minimally invasive implants permit vertebral body instrumentation with different screw types. Apart from different screw diameters, the coupling interface to shaft of the screw is of crucial importance. Monoaxial, unilateral and polyaxial screws are available. In some systems these different screw types are offered in different options allowing for bone cement augmentation.

In clinical routine, amongst other things, the primary stability of pedicle screws depends on their positioning and alignment. Notably, special care should be taken of the cranio-caudal pedicle screws positioning [24].

The susceptibility to screw loosening is influenced by the indications for pedicle screw fixation and the bone tissue quality. Loosening appears to be a minor clinical problem for fixation and fusion of healthy, non-osteoporotic bone [25].

The aim of the present study was a biomechanical comparison of different posterior spine instrumentation systems to investigate the influence of the screw design and the associated variations of the connection to its shaft on the axial and torsional stiffness, as well as on the number of cycles to failure.

In the current study, the plain radiographs showed that axial loading of the USS led to higher proximity of the two foam blocks together with the implanted screws. This was due to plastic deformation of the longitudinal rods and, consequently, due to the relative torsion between the two bars, with associated slippage of the connectors between the Schanz screws and the longitudinal rod. Macroscopic and radiological analysis showed that the screw-to-foam-bar interface remained unchanged.

In contrast, the constructs with monoaxial screws showed breakage or screw pull-out from the foam bar. One possible reason for these types of failure might be the fact that it is very difficult to precisely align the side openings in the respective two screw heads for placement of the longitudinal rod. Wang et al. have reported that small discrepancies in positioning and fixation of the screw-rod system with monoaxial screws may give a rise to intervertebral translational and rotational forces with negative effects at the screw-to-vertebral-body interface [26]. In turn, this may lead to screw loosening or cut-out [27].

Polyaxial screws were found to fail in the region of the screw head. This finding is consistent with the mechanical weakness of these screws reported in the literature. In a biomechanical study on polyaxial pedicle screws, Fogel et al. reported that the connection between the screw head and shaft failed first. They also stated that the lesser stiffness of the coupling mechanism might lead to reduction in bending stress and therefore prevent screw cut-out or screw failure [14]. Stanford et al. also reported that polyaxial screw heads and their multiaxial link are vulnerable to fatigue failure [28]. However, this failure generally results in correction loss or, possibly, loss of reduction and height of the vertebral bodies in the sagittal profile.

The results from the present study also showed lower torsional stability of the minimally invasive systems compared to USS, whereby axial bending stiffness was comparable between the study groups. Polyaxial instrumentation failed earlier in the destructive test compared to both monoaxial constructs and USS. In our opinion, this failure has to be attributed to the coupling mechanism between the polyaxial screw head and its shaft.

Based on the current findings, prevention of early correction loss and/or reduction loss in the management of unstable fractures of the spinal column requires stable fixation with a conventional open locked implant such as the USS or a minimally invasive system with monoaxial screws.

In summary, the spinal column is a complex structure with loading patterns that are difficult to mimic biomechanically. This is equally true for artificially created pathologies. Further studies should evaluate the screw-to-vertebral-body interface taking into account differences in bone quality, especially considering osteoporotic bone. Testing of a wider range of systems in larger samples of specimens would be desirable along with evaluation of augmentation procedures in cadaveric studies.

Uniplanar screw designs seem promising in this context since they combine the advantages of polyaxial and monoaxial systems.

It has to be admitted that the results in this study might have been influenced by some simplifications of the biomechanical model used. It would be desirable to test comparable screw lengths and diameters as well as longitudinal rods of comparable diameters to achieve better statement validity. In addition, differences between the implant positions and orientations may have influenced the interacting forces. However, such kind of errors have to be considered in all fracture models and therefore, this comparative study provides valuable information on the differences between the investigated spine instrumentation systems.

Supplementary, there is a need of further (in vivo) studies to transfer the findings to clinical routine.

## Conclusion

The results of the current study quantify for the first time the loss of correction reported in the literature for minimally invasive polyaxial screw systems.

Specifically, the use of polyaxial screws for stabilization of unstable fractures or defects of the anterior column in inflammatory disease or spine tumors should be evaluated carefully, because the risk of renewed subsidence with consequent deformity of the sagittal profile is seems to be considerably higher.

## Abbreviations

ANOVA: One-Way Analysis of Variance
USS: Universal Spinal System.
